# A Comparison of Two Macroinvertebrate Multi-Plate Sampling Methods to Inform Great Lakes Monitoring and Remediation Efforts

**DOI:** 10.4236/jep.2023.1412052

**Published:** 2023-12-06

**Authors:** Roger Yeardley, Brian Duffy, Kimani Kimbrough, Jim Lazorchak, Marc A. Mills, Ed Johnson

**Affiliations:** 1 Office of Research and Development, United States Environmental Protection Agency, Cincinnati, USA; 2 New York State Department of Environmental Conservation, Albany, USA; 3 National Ocean Service, National Oceanic and Atmospheric Administration, Silver Spring, USA

**Keywords:** Hester-Dendy, Multi-Plate Samplers, Macroinvertebrate, Bioassessment, Great Lakes, Methods, Area of Concern

## Abstract

Hester-Dendy (HD) multi-plate samplers have been widely used by state and federal government agencies for bioassessment of water quality through use of macroinvertebrate community data. To help guide remediation and restoration efforts at the Niagara River Great Lakes Area of Concern site, a multi-agency study was conducted in 2014 to assess the contribution of seven major urban tributaries on the US side of the river toward the impairment of the Niagara River. As part of this study, macroinvertebrate communities were sampled using two co-located versions of HD samplers: one version used by the New York State Department of Environmental Conservation (NYSDEC) and another by the US Environmental Protection Agency Office of Research and Development. Samplers were deployed in tributaries in highly developed watersheds with high percent impervious surface. The two sampling methods varied in terms of number and size of plates, between-plate spacing, and deployment method. Comparison of the similarity/grouping of communities with multivariate ordination techniques, Nonmetric Multidimensional Scaling and Multi-Response Permutation Procedure, showed that both methods were able to detect differences in communities at stations, despite some grouping by month and method. The indices and metrics derived from the two HD methods were found to give comparable but not identical assessments of water quality. Despite their differences, the methods were robust with respect to water quality categories derived from indices used nationally (HBI) and by NY state (BAP). For the common richness metrics, total taxa and EPT richness, there was no statistical difference between means from 3 samplings. Some metrics, especially percent tolerant collector-gatherer individuals, did show significant differences at certain stations. Indicator Species Analysis showed some taxa associated with each method. The observed community differences were thought mostly due to the difference in sampler deployment position.

## 1. Introduction

In 1972, Environment Canada (EC) and US Environmental Protection Agency (USEPA) established the Great Lakes Water Quality Agreement and committed to restoring the physical, chemical, and biological integrity of the Great Lakes [1]. This agreement and subsequent amendments developed a framework to promote the ecological health of the Great Lakes. In 1987, EC, USEPA, the Ontario Ministry of the Environment and New York State Department of Environmental Conservation (NYSDEC) signed the Niagara River Declaration of Intent to adopt and implement a toxics reduction plan [2]. The main stem of the Niagara River was designated as an Area of Concern (AOC) based on seven beneficial use impairments (BUIs) identified: 1) restrictions on fish and wildlife consumption, 2) fish tumors or other deformities, 3) degradation of benthos, 4) restriction on dredging activities, 5) loss of fish and wildlife habitat, 6) degradation of fish and wildlife habitat, and 7) bird or animal deformities or reproductive problems [3] [4]. The seven BUIs were driven by historical, industrial, and municipal discharges, waste disposal, and impacts to ecology, stemming from PCBs, mirex, chlordane, dioxin, hexachlorocyclohexane, and polynuclear aromatic hydrocarbons [3] [4]. The Niagara River AOC is a highly developed area impacted by legacy contaminants and Contaminants of Emerging Concern, impervious surfaces, and high population density [5] [6].

In 2014, a multi-agency study was conducted at the Niagara River AOC in Buffalo, New York, to characterize the relative impacted states of AOC tributaries and identify contaminant sources. The interagency, transdisciplinary team consisted of scientists from the USEPA Office of Research and Development, National Oceanic and Atmospheric Administration, NYSDEC, United States Army Corps of Engineers, the University of Wisconsin-Milwaukee, and others. Accurate monitoring and assessment of water quality at contaminated sites like Niagara River AOC are critical to provide useful data to stakeholders, provide baseline data, establish remedial prioritization, and to determine the effectiveness of remedial efforts. The data are being used in a multiple lines of evidence, weight-of-evidence approach to provide baseline information on the extent that beneficial uses are impaired in order to measure future cleanup progress [6].

As part of the Niagara River AOC characterization study, and as part of a long-term commitment of USEPA to provide information to states and USEPA regions on effective monitoring techniques, a methods comparison study was conducted on two versions of a Hester-Dendy (HD) multi-plate artificial substrate sampler. Multi-plate samplers/HDs have been used by many states [7]–[14] and some federal agencies [15] in the US, as well as worldwide [16] for biological monitoring of the quality of surface waters. HD design (plate size, spacing), number of HDs, and deployment location can vary between states. Hereafter samplers will be referred to as EPA and NY HDs.

Multi-metric indices are recommended by USEPA [14] as an important part of state bioassessment programs to “strengthen data interpretation and reduce error in judgement based on isolated indices and measures.” The NYSDEC utilizes a multi-metric index of water quality known as the Biological Assessment Profile (BAP) score [10]. Component metrics include species richness, Ephemeroptera, Plecoptera, and Trichoptera (EPT) richness [17], Hilsenhoff’s biotic index (HBI) score [18], and Shannon-Wiener diversity, and are normalized on a 0 - 10 scale and averaged to calculate the BAP. Indices like the BAP and HBI, beyond just assessing the similarity of communities retrieved, also incorporate other qualities (e.g. pollution tolerance) of the organisms retrieved, that give additional information on the water quality or station/site impact. Different types of metrics and indices have also been shown to be useful measures of water quality. US state programs use a mixture of metrics focused on structural taxa richness, tolerance values (*i.e.*, measures of sensitivity), and functional or ecological attributes of macroinvertebrates. Though not generally considered sufficient as stand-alone measures of water quality [19], taxa richness metrics are commonly used by states as part of their bioassessment programs [10] [14] [19].

Analyses of macroinvertebrate communities collected with HDs have proven useful for monitoring and assessing water quality and ecological health of a wide range of waterbodies, including those being remediated as part of the Great Lakes Restoration Initiative [20]. Understanding methodological differences is necessary for understanding comparability of various sampling methods employed across the 43 Great Lakes AOCs, and for understanding how methodological differences affect monitoring and assessment results. The objectives of this study were to: 1) determine if both methods were able to detect differences in macroinvertebrate communities at stations within this Great Lakes AOC site, 2) to determine the comparability of index rating categories of water quality/impact derived from each method, and 3) the comparability of individual metric values.

## 2. Materials and Methods

### 2.1. Study Area

**Figure 1.**
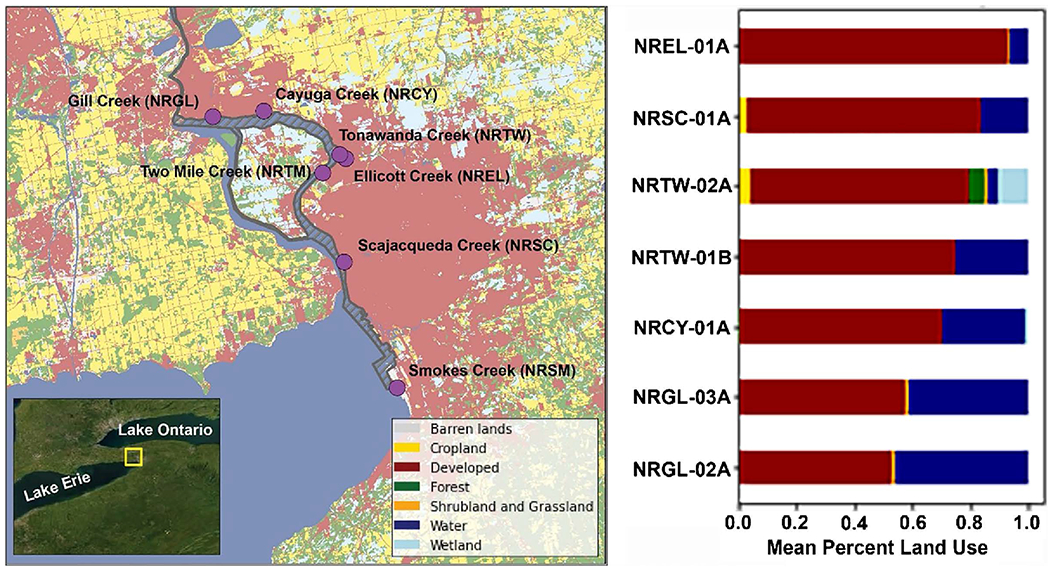
Study Area—Stations and Land Use. Map (left) showing station locations and land use affecting urban tributaries sampled at the Niagara River Area of Concern. Thatched area shows extent of the AOC. Graph (right) shows percent land use within 1-km buffers surrounding each station.

A sampling campaign on six U.S tributaries of the Niagara River (Figure 1) was conducted to assess their relative contributions to impairment of the Niagara River AOC and collect data to guide remediation efforts. Sampling was intended to characterize pre-remediation conditions and consisted of physical, chemical, and biological characterization of the upper Niagara River and its tributaries.

**Figure 2.**
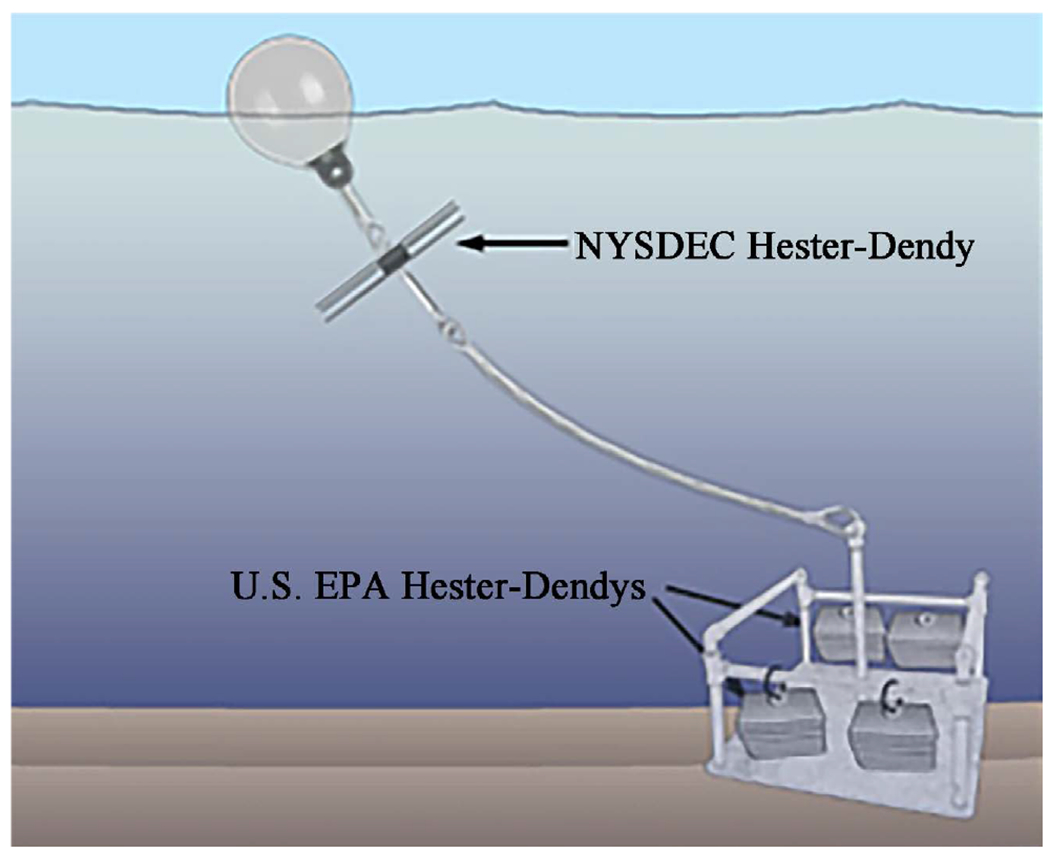
Deployment of New York and EPA HD Samplers. NY samplers float in the water column 1 meter below the surface. EPA HDs are attached to moorings placed on the sediment surface. Actual number of EPA HDs (12), not shown.

At each station on a tributary, multiple sampling devices were attached to moorings that collected data on contaminant concentrations [6] and macroinvertebrate communities. Among these sampling devices were co-located (Figure 2) NY and EPA HDs, which were deployed at eight stations on six Niagara River AOC tributaries: Two Mile Creek, Scajaquada Creek, Ellicott, Gill Creek (two stations), Tonawanda Creek (two stations), Smokes Creek, and Cayuga Creek (Figure 1, Table 1). Using data from the North American Land Change Monitoring System [21], an analysis of the area within 1-km buffers of the stations was conducted to characterize the land use around the stations (Figure 1).

### 2.2. Construction and Deployment of Samplers

**Figure 3.**
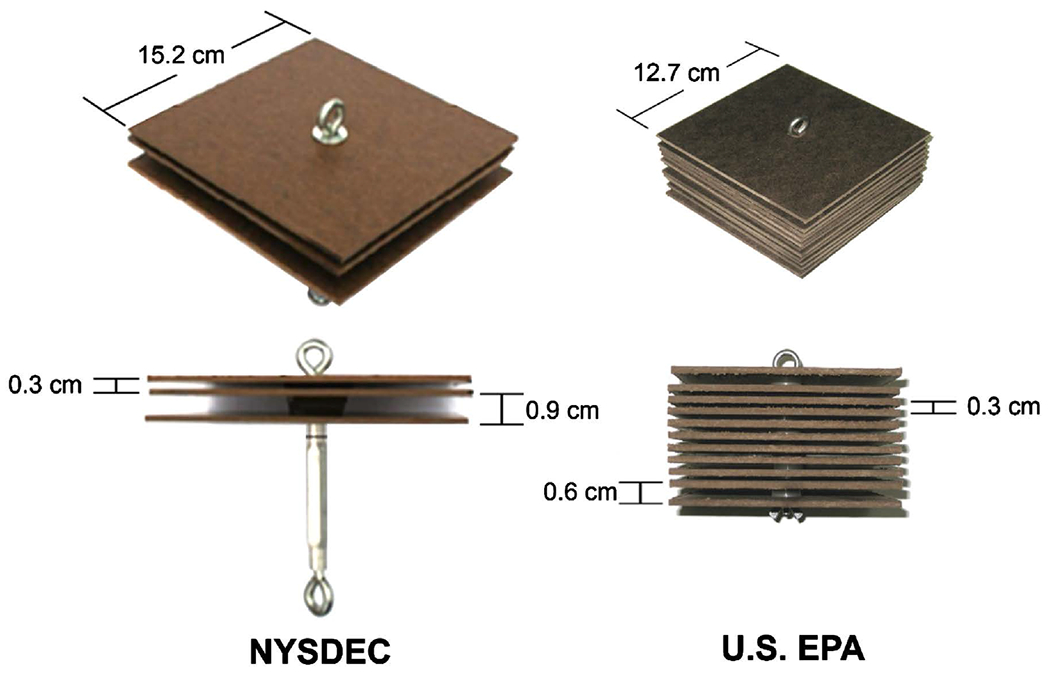
Design of New York State DEC (1386 cm^2^) and EPA (3548 cm^2^) HD samplers.

Both versions of HD samplers were constructed with tempered hardboard plates mounted on threaded eyebolts. NY HDs were constructed with three 15.2-cm × 15.2-cm (6-inch × 6-inch) plates separated by 0.3 cm and 0.9 cm [10] and a total surface area for each sampler of 1386 cm^2^. EPA HDs had eleven 12.7-cm × 12.7-cm (5-inch × 5-inch) plates with between-plate spaces of 0.3 cm and 0.6 cm, and a total surface area for each sampler of 3548 cm^2^ (Figure 3). Each sample represented the macroinvertebrates retrieved from one (NY method) or two (EPA method) HDs. The NY HD samplers were suspended 1 meter below the water surface [10] [22] and anchored to the mooring (Figure 3). EPA HD samplers were deployed 20 cm above the sediment by attachment to a mooring placed on the sediment surface. All HD samplers were deployed for a five-week colonization period.

### 2.3. Hester-Dendy Apparatus Retrieval and Processing

HDs were retrieved in July, August, and September after five-week deployments. Unless otherwise indicated, the separate deployments are represented as the average of these three retrievals in the analyses. Each mooring was raised from the sediment surface to the deck of the boat, and the HD samplers were removed from the mooring and submerged in water from the sampling locations and transported to shore. Once on shore, each HD was disassembled, and the plates scraped of all debris and organisms. The sample was collected by passing the contents through a 500 μm sieve with repeated rinses to remove debris.

For both NY and EPA HDs, all contents collected on the sieves were preserved in 70% ethanol for sample processing. For sample sorting and ID, NY Standard Operating Procedures [10] were used for samples collected from both NY and EPA HDs. For sorting, the contents from each HD were divided into quarters and sequentially processed in their entirety until the target subsample count of 250 organisms was achieved (or exceeded) or until the entire sample had been processed. Macroinvertebrates were identified to the lowest practical taxonomic resolution (usually genus or species). The first 50 and 100 organisms in the *Oligochaeta* and *Chironomidae* groups, respectively were slide mounted and identified, beyond which additional organisms in these groups were assigned identifications proportionately, following NYSDEC Standard Operating Procedures [10].

### 2.4. Data Analysis

#### 2.4.1. Indices

We compared the results of Hilsenhoff Biotic Indices calculated from the derived from tolerance values from the literature [23] and macroinvertebrate taxa data from the EPA and NY HDs. The HBI represents the average weighted pollution tolerance value of all macroinvertebrates present in a sample [18]. Taxa tolerance values range from 0 to 10, with 0 being most sensitive or intolerant and 10 being most tolerant. We also made comparisons using variations on the HBI, the 10-Max BI (HBI_10_) and mean tolerance value (MTV). For the HBI_10_, a later refinement of the index, Hilsenhoff [24] [25] recommended that when a sample had more than 10 individuals of any taxa, only 10 be used in calculation of the index. The HBI_10_ was meant to lessen the effects of large numbers of tolerant taxa, which can sometimes be found in undisturbed streams and are frequently found in polluted streams. The MTV metric represents an average of the tolerance values of all taxa present at a station and does not consider taxa abundance. The MTV metric also lessens the effects of large abundances within tolerant taxa tolerant taxa.

We compared the NYSDEC multi-metric BAP index for communities retrieved from the NY and EPA HDs. To calculate the BAP, component metrics, including species richness, EPT richness [17], HBI score, and Shannon-Wiener diversity, are normalized and ranked on a scale from 0 to 10, and averaged to calculate the BAP. The BAP is divided into four impact categories: non (7.5 - 10), slight (5.0 - 7.5), moderate (2.5 - 5.0), and severe (0 - 2.5) impact. BAP scores were calculated from the raw data of taxa retrieved from each HD version.

Our criteria for considering the water quality rating or impact category from both methods to be in the same category were 1) if both means fell into the same category (whether or not t-tests showed there to be a statistically significant difference between them, or 2) if both means didn’t fall into the same category, but t-tests showed there was not a statistically significant difference between them. If means fell into different categories and there was a statistically significant difference between them, we considered them to be in different water quality categories.

#### 2.4.2. Metrics

For comparison of the water quality ratings (based on macroinvertebrate assemblages) retrieved by the two methods, we chose eight metrics that assess diversity, tolerance, and functional feeding group composition of the communities. Three of these were richness metrics, involving number of taxa from different groups—total taxa richness, EPT richness, and insect richness. The fourth metric, number of collector-filterer (C-F) taxa, represented functional feeding group information. The fifth and sixth metrics represent combined tolerance and functional feeding group characteristics: number of tolerant collector-gatherer taxa and percentage of tolerant collector-gatherer individuals. Abundance and density were the seventh and eighth metrics. Density was calculated by dividing abundance by the total surface area (top and bottom) of the HD plates.

#### 2.4.3. Statistical Analyses

##### 1) Multivariate Ordination of Ecological Communities

To compare similarity of macroinvertebrate communities, Nonmetric Multi-dimensional Scaling (NMDS) ordinations, along with Multi-Response Permutation Procedure (MRPP) analyses were conducted and graphs generated (PC-ORD^™^ 7) from matrices of taxa abundance data. NMDS plots provide a visual representation that shows if grouping is occurring. NMDS analyses were run on non-transformed data with rare taxa removed, using a Sorensen distance measure and the PC-ORD^™^ autopilot function, and accepted the suggested solution (*i.e.*, the one with the least stress), which was three-dimensional in most cases. MRPP, which generates test statistics that indicate the degree of difference between groups, was performed on the data matrices after the NMDS analyses.

For the NMDS, PC-ORD^™^ finds the best positions of *n* entities (samples) on *k* dimensions (axes) by doing an iterative search that minimizes the stress of the final configuration. Smaller stress means better fit. The MRPP analysis provides test statistics indicating the amount of separation between groups. The test statistic T describes the differences between groups by comparing an observed delta to an expected delta. The more negative T is, the stronger the separation [26] of the average within-group distance. The calculated expected delta represents the mean delta for all possible partitions of the data. The agreement statistic, A, is the chance-corrected within-group agreement or homogeneity. In community ecology, values for A are commonly below 0.1 [26]. An agreement statistic equal to 1 (A = 1) indicates that all items are identical with groups. The p value is used to evaluate how likely an observed difference is due to chance or the likelihood of getting a delta (the average within-group distance) as small, or smaller than the observed delta.

The Indicator Species Analysis [26] combines information on the concentration of taxa abundance in a particular group and the faithfulness of occurrence of a taxon in a particular group. Here, the version of this analysis in PC-ORD^™^, is used to describe taxa relationships to the two experimental groups of NY and EPA HDs.

##### 2) t-tests

To compare metric and index results One-way paired t-tests were run in Sigmaplot 14 to determine where statistically significant differences existed (p ≤ 0.05) between for the two methods. For instances where Shapiro-Wilk normality or Brown-Forsythe equal variance tests failed, a Mann-Whitney Rank Sum Test was used.

## 3. Results

### 3.1. Community Similarity Analyses

**Figure 4.**
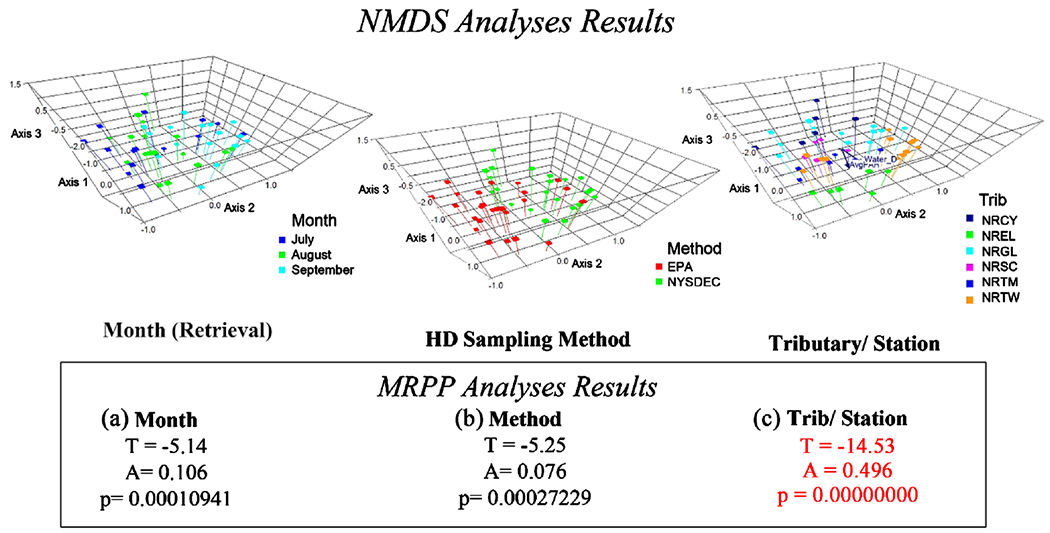
Results of NMDS analyses, showing grouping by (a) Retrieval month, (b) Sampling method, and (c) Tributary, along with results of MRPP analyses, showing statistical evidence of grouping with strongest grouping by tributary/station. NMDS stress = 14.6. For MRPP analyses, smaller T = stronger grouping (McCune and Grace 2002).

NMDS and MRPP analyses indicated that some grouping occurs by month and HD method but there is a strong grouping by tributary (Figure 4). Therefore, both HD methods were able to distinguish station/tributary differences in macroinvertebrate community makeup.

**Figure 5.**
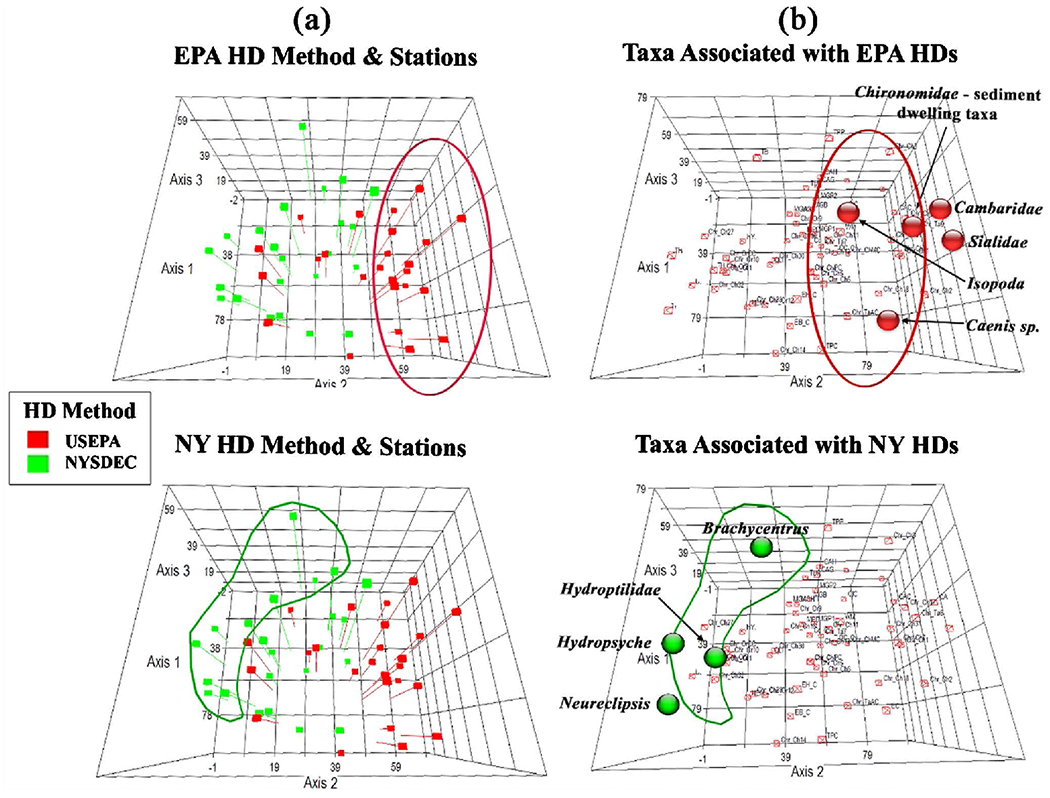
NMDS graphs were able to tease out some differences between communities retrieved by each method and showed (a) grouping of communities associated with NY or EPA HD methods, (b) Some taxa associated with the communities retrieved by each sampling method. Stations plotted in species space; stress = 14.6.

Using NMDS to plot points for both stations and taxa shows that there is some grouping of communities by sampling method (Figure 5(a)), and that certain taxa are predominantly associated with one method (Figure 5(b)). The NY HDs, which are deployed closer to the water surface, retrieved more net-spinning, filter feeding caddisfly genera (*Hydropsyche*, *Neureclipsis*, *Brachycentrus*), all of which need a moderate current to capture food [27], and *Hydroptilidae*, which prefer algae and macrophytes. EPA HDs, which are deployed in closer proximity to the sediment, retrieved more sediment-dwelling taxa (*Cambaridae*, *Sialidae*, *Isopoda*, *Caenis*, and several *Chironomidae* genera) (Figure 5(b)).

An Indicator Species Analysis [26] (Table 2) also showed that certain taxa were associated with each HD method. There were eight taxa primarily associated with the EPA samplers and one taxon associated with the NY sampler.

### 3.2. Comparison of Indices’ Water Quality Ratings

**Figure 6.**
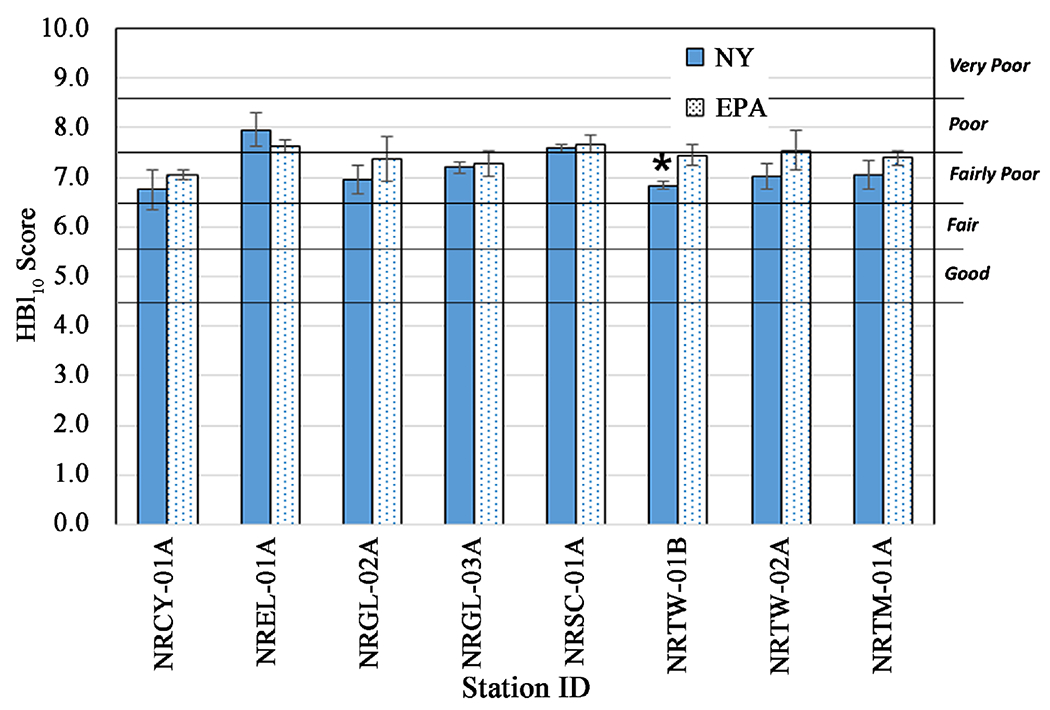
Comparison of Hilsenhoff Biotic Indices (HBI_10_, limiting each taxon to a maximum of ten individuals) calculated from taxa retrieved by NY and EPA HD methods. Water quality categories corresponding to score intervals from Hilsenhoff (1987). Error bars represent the standard error of values from three months of sampling. ⋆ = statistically significant difference (*p* ≤ 0.05) and indicating the method showing better water quality.

Based upon the criteria laid out in section 2.4.1, the modified Hilsenhoff Biotic Index (HBI_10_) water quality rating categories (based on the mean scores of the 3 deployments (July, August, September), were the same for all eight stations (Figure 6). For five of the eight stations, both methods returned a water quality rating of Fairly Poor (6.51 - 7.50). For two stations both methods gave a Poor (7.51 - 8.50) rating (Figure 6). For station NRTW-02A, the category is a bit more ambiguous, though our criteria say it is the same category for both methods. The HBI_10_ mean score of 7.54 generated from the EPA HD community data is just barely in the Poor rating category, while the mean score of 7.02 generated from the NY HD community data put this station solidly in the Fairly Poor category.

**Figure 7.**
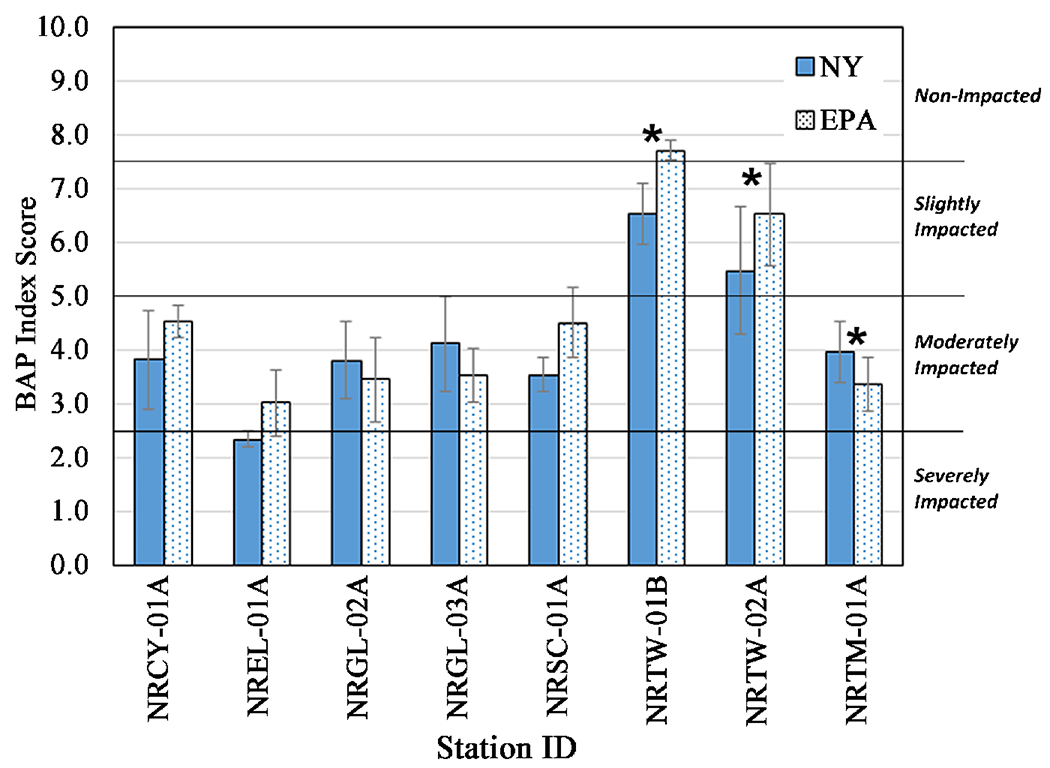
Comparison of Biological Assessment Profile (BAP) Indices calculated from taxa retrieved by NY and EPA HD methods. Error bars represent the standard error of values from three months of sampling. ⋆ = statistically significant difference (*p* ≤ 0.05) and indicating the method showing better water quality.

For the multi-metric index comparison, mean BAP values of each station were categorized into one of four index categories of impact (Figure 7). Based on our criteria, impact categories derived from the two methods were the same for seven of eight stations. Five stations were clearly in the Moderately Impacted category (2.5 - 5 on the BAP scale) regardless of method, and one station was rated as Slightly Impacted (5 - 7.5 on the BAP scale) by either method. One station (NRTW-01B) which had a statistically significant difference in mean values, was in the Slightly Impacted category according to the NY method and in the Non-Impacted category according to the EPA method. One station (NREL-01A) would be considered ambiguous regarding category by either method (Figure 7), on the border of Moderately and Severely Impacted.

Comparisons of categories of the BAP values by each month were also done (Table 3). Comparing categories by the strict calculated values, not allowing for any variability, for 15 of the 23 station samplings for which we had 2 data points (for we only had one), the 2 methods generated the same impact category. Some of these values were very close to being on the border between 2 categories. For example, at site NRTM-01A, the NY method generates a value of 5.06, just barely in the Slightly-Impacted category, while at 4.23 the EPA value is within the Moderately Impacted category (Table 3). If we allow for a variability of ±0.1 for all values, 20 of the 23 comparisons (or 87%) could be said to yield the same impact category.

### 3.3. Comparison of Metric Values

**Figure 8.**
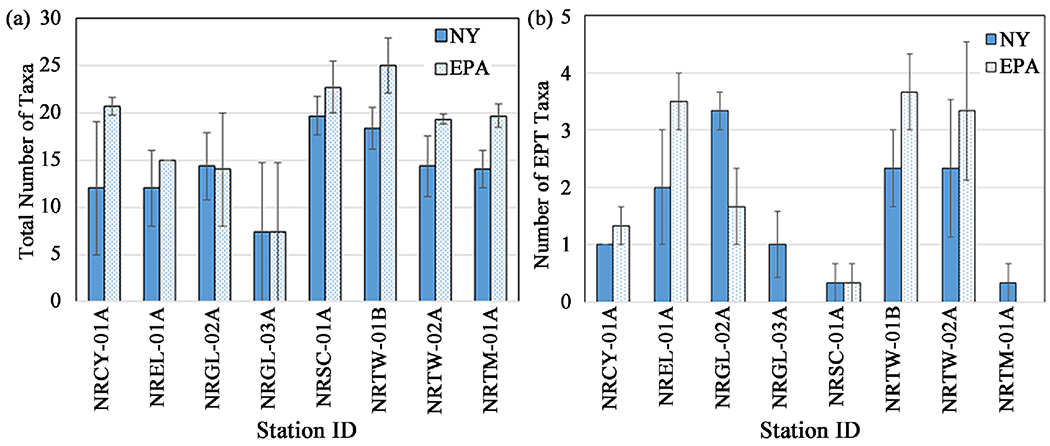
Taxa Richness metrics—Comparison of number of taxa retrieved by NY and EPA HD methods (a) Total taxa richness; (b) Ephemeroptera-Plecoptera-Trichoptera (EPT) taxa richness. Error bars represent the standard error of values from three months of sampling.

**Figure 9.**
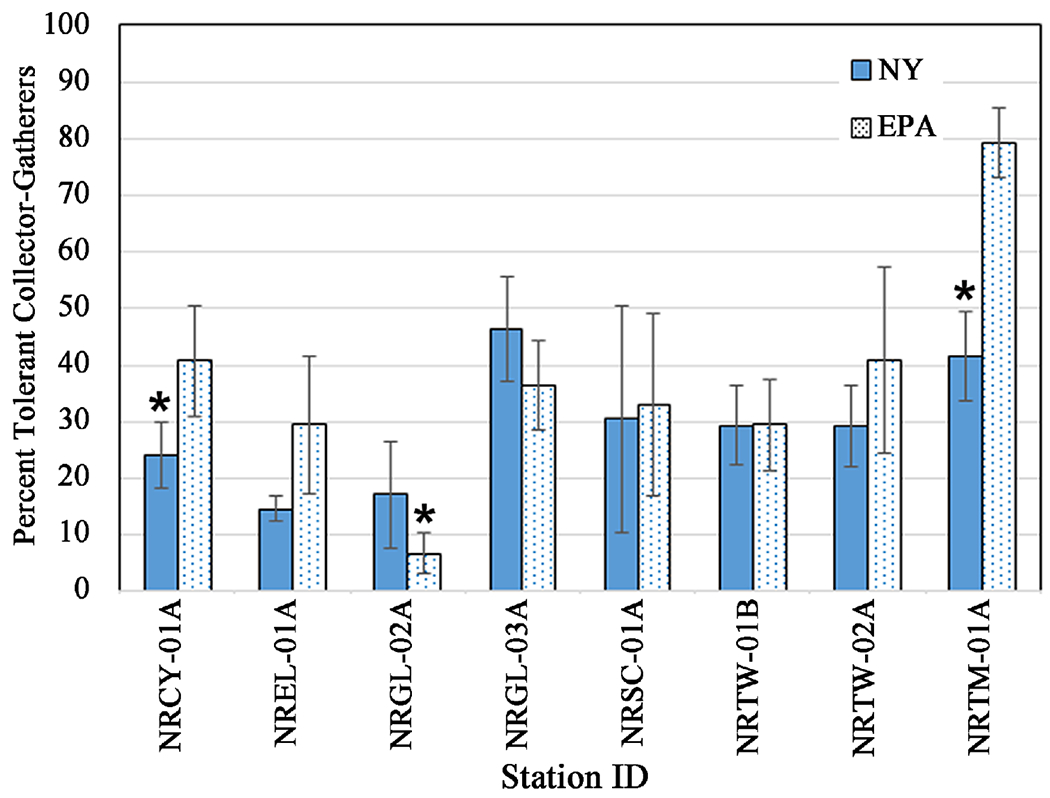
Percent of tolerant Collector-Gatherers (C-G) collected by each HD method. Error bars represent the standard error of values from three months of sampling. ⋆ = statistically significant difference (*p* ≤ 0.05) and indicating the method showing better water quality.

**Figure 10.**
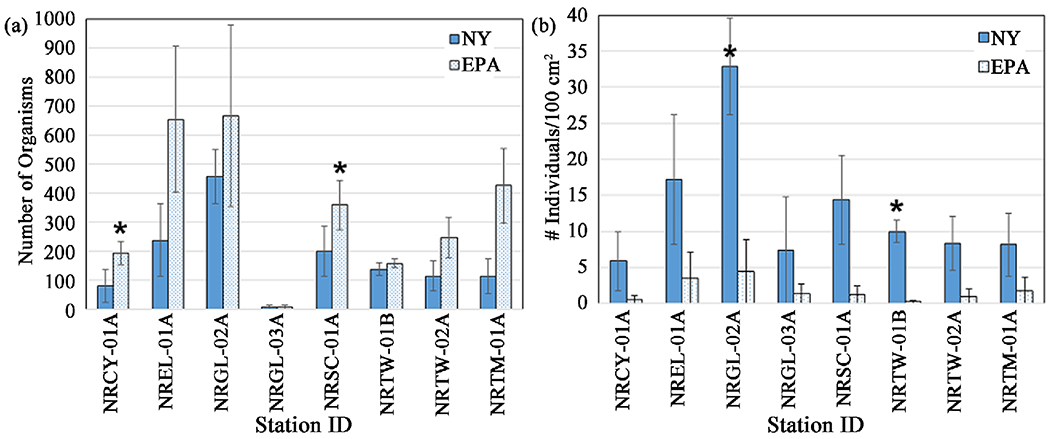
(a) Total abundance of macroinvertebrates collected by New York and EPA HD sampling methods (two EPA HDs vs one NY HD). (b) Densities of macroinvertebrates retrieved by each method expressed as abundance per 100 cm^2^. Total numbers retrieved by each method divided by plate area of each method where the surface areas equaled 1386 cm^2^ and 7096 cm^2^ for the NY method and the EPA method, respectively. Error bars represent the standard error of values from three months of sampling. ⋆ = statistically significant difference (*p* ≤ 0.05).

For consecutive 5-week deployments starting in July, August, and September, at eight stations on tributaries in watersheds with a majority of land use categorized as “developed” (Figure 1), we compared metrics derived from the two methods. The two HD methods did not differ in total taxa richness and EPT richness retrieved at all stations (Figure 8, Table 4). They also did not differ in number of collector-filterer taxa or collector-gatherer taxa (Table 4). For percent tolerant collector-gatherer individuals, there was a significant difference between methods at two stations (Figure 9, Table 4), NRCY-01A and NRTM-01A, with the EPA method collecting a higher percentage of C-G individuals. The two HD methods retrieved abundances and densities of macroinvertebrates that were not statistically significantly different at six of the eight stations (Table 3). At two stations (NRCY-01A and NRSC-01A), the EPA HDs retrieved a greater abundance of macroinvertebrates (Figure 10(a)). At two stations (NRGL-02A and NRTW-01B), the NY HDs retrieved a greater density of macroinvertebrates (Figure 10(b), Table 4).

## 4. Discussion

### 4.1. Sources of Variability

It should be noted that in using standard statistical methods to examine potential method similarities and differences regarding macroinvertebrate indices and metrics (Table 2), a couple of factors limit the power to detect differences. First, having a relatively small sample set of eight stations somewhat limits this power. The variability of the data at each station (e.g., represented by the error bars in the histograms in Figures 5–9) is also a factor in being able to detect differences between stations, and would be expected to be influenced by the temporal differences introduced by the three samplings during different months. But the fact that within-month comparisons of impact categories derived from the two methods also confirmed this robustness, is evidence that this added variability is not unusually high.

Seasonal variability in macroinvertebrate communities is a well-known phenomenon. This knowledge contributes to the USEPA usually sampling at AOC sites during a fall index period. Regarding seasonal variability, Hilsenhoff [25] asserted that in late spring and in summer streams are warmer with less oxygen, and that some intolerant/sensitive species will spend this period as eggs or in diapause. According to this paradigm, there would be a lower number of sensitive taxa and therefore more tolerant taxa available for sampling in the warmer summer months. For these reasons Hilsenhoff [18] recommended that HBI use be restricted to spring and autumn. Though percent tolerant organisms showed no difference by month, where we have seen statistical differences in metrics by month, such as percent C-G and total taxa number, better water quality was seen in September. The highest BAP values were often seen in September (Figure 3). All but one of the samplings with a low number of taxa (< 10) are in July and August (likely the hotter months), with seven of ten of these being in August. The average number of species for each type of sampler was lowest in August (EPA = 14.5, NY = 10.8), and highest in September (EPA = 19.5, NY = 16.9). Unfortunately, we do not have dissolved oxygen data for the various stations and deployment periods to be able to test differences in oxygen levels as a likely cause for the community differences seen between deployment periods.

An area in which there was not a great variability between stations was in levels of development. An analysis of land use [21] in the area within 1-km buffers around the sites (Figure 1) showed high levels of development indicative of urban/developed land use. Percent impervious surface was greater than 30% at all stations. The situation at the Niagara River is typical of Great Lakes AOC sites, where watersheds or hydrological unit codes (HUCs) of all study sites are typically highly developed with a high percentage of impervious surface. As a result, there is a dearth of unimpacted stations available for use as reference. Therefore, there is a need to develop new approaches, and to find metrics and indices that can distinguish levels of impact among stations in highly developed, impacted watersheds. For example, an ambient distribution approach [28], which utilizes percentile values of a distribution of metric values to rank stations by relative impact can be used. To use such an approach, metrics and indices that are sensitive to station differences are necessary.

### 4.2. Choice of Metrics and Indices

For our comparisons of metrics derived from each HD method we chose four metrics which are used by a large percentage of US state environmental agencies. A survey of stream bioassessments performed by US State Agencies [29] found richness and percent composition metrics to be the most-commonly used. The two most-commonly employed were total richness (60%; 28 of 47 states) and EPT richness (57%; 27 of 47 states). The metric used most by state programs was total taxa richness, which was considered the most useful metric by 43% of state respondents. Lenat [17] also reported EPT richness as a useful and commonly used metric. Weigel and Dimick [13] found that EPT taxa richness had a strong inverse correlation with disturbance. We also included abundance and density metrics, which are commonly used, but less so than richness metrics [29].

Regarding the less commonly used metrics that we chose, Weigel and Dimick [13] found that insect taxa richness had one of the highest inverse correlations with disturbance of 47 metric variations that they evaluated. Metrics that utilize tolerance values and/or functional feeding group (FFG) information are used by 96% of US state bioassessment programs [10] [29], and world-wide [16] [30]. Though not widely used, Weigel and Dimick [13] found that percent tolerant collector-gatherers (C-G) metric had a high correlation with disturbance. This metric has the advantage of combining FFG and tolerance characteristics of the biota. We also included number of tolerant C-G taxa. Number of collector-filterer (C-F) taxa is another important functional feeding group that can correlate with level of flow at a station.

### 4.3. Multivariate Analyses-Community Similarity and Ability to Detect Community Differences

It is important for any monitoring and assessment method to be able to detect temporal and spatial differences. For Great Lakes AOC sites, it is especially important to be able to detect differences between stations, and between pre- and post-remediation and restoration. Analyzing an ability to detect differences in community ecological data consisting of taxa abundance data from multiple species, involves a higher degree of difficulty than, say for contaminant concentration data. Thus, special tools are useful. Multivariate NMDS graphs were able to show some temporal differences (deployment month) in communities and differences by sampling method and (Figure 4, Figure 5). NMDS and ISA analyses showed some organisms more closely associated with one method. However, statistical MRPP analysis (Figure 4) showed a much stronger grouping of communities by station or tributary, than by sampling method or deployment month, indicating that either method would be capable of detecting differences in communities (and therefore in water/sediment quality) between stations. This strong grouping by location indicates that macroinvertebrate community data from either method may be used in weight of evidence assessments along with contaminant body burden data, and other site physical, chemical, and biological indicators, to rank stations or tributaries in terms of degree of impact, and thus can be useful in guiding remediation and restoration efforts.

### 4.4. Water Quality Assessment-Similarities of Method Results

Considering differences between the two monitoring and assessment methods, including different plate size and spacing and location in the water column, the multi-plate artificial sampler methods examined were fairly robust in giving similar overall water quality and impact assessment categories of each station, derived from indices. Multi-metric indices are the primary means of assessing water/sediment quality used by multiple Great Lakes states [10] [11] [12]. The majority of stations showed no difference for the BAP multi-metric index values and a sizeable majority (6 - 7 stations) showed no difference in value for HBI-related indices (HBI, HBI_10_, MTV) that incorporate tolerance values (Table 3, Figure 7, Figure 8).

Though the metrics generated from the data on macroinvertebrate communities retrieved by the two types of HD samplers were comparable, the comparisons were not quite as robust as for the impact categories. For four of the eight metrics, including two of the most widely used metrics, total taxa richness and EPT richness, no significant differences were seen at any stations (Table 3, Figure 5).

Currently, the USEPA is conducting studies to examine if this robustness seen in this study also exists between indices and metrics derived from the EPA HD sampling method and HD methods with 3-inch × 3-inch plates utilized by Great Lakes states Ohio [11] and Wisconsin [12], as well as with other variations on multi-plate samplers [31].

### 4.5. Water Quality Assessment—Differences in Method Results

Despite the robustness of the multi-plate samplers with respect to water quality categories, and a number of metric and index values, due to the multiple differences in the methods, they would not be expected to retrieve exactly the same macroinvertebrate communities, especially as they are deployed at different depths in the water column. The stations which showed the most differences between metrics and indices for the two methods, NRTW-01B (two metrics, two indices) and NRTW-02A (three indices) (Table 3), were also the two deepest stations (NRTW-01B = 5.2 m, NRTW-02A = 3.2 m (Table 1)), with the largest distances between the two sampler versions. Multivariate ordinations for ecological communities, NMDS (Figure 4, Figure 5) and Indicator Species Analysis (ISA) (Table 2), revealed that in addition to a number of taxa common to both co-deployed sampler versions, certain taxa were predominantly associated with one HD method. These analyses also support a hypothesis that the difference in habitats offered by the two sampler versions (primarily based on position in the water column) is likely a factor in the differences seen in communities retrieved on the methods. Two organisms that were strictly benthic were associated with the EPA HDs, which were deployed on the sediment surface. *Cambaridae* (crayfish), was both statistically strongly associated with EPA samplers and only found on EPA samplers. The benthic taxon *Caecidotea* was also associated with EPA HDs (Table 2). *Sialis* and *Cladopelma*, which prefer a depositional habitat, though not found at enough stations to detect a statistical difference (p was not < 0.05) in ISA, were only found on EPA HDs. Several *Chironomidae* taxa that prefer depositional habitat [32] were strongly associated with the EPA samplers, while no chironomid taxa were found to be specific to the NY samplers (Table 2).

Correlations were found between NY HDs and collector-filterer taxa and those that prefer a faster flow. Three genera of Trichoptera (*Hydropsyche*, *Neureclipsis*, *Brachycentrus*) that prefer faster flow were found through NMDS analysis (Figure 4) to be associated with NY HDs. Two of these genera (*Hydropsyche*, *Brachycentrus*), though not found at enough stations to detect a statistical difference in ISA were only found on the NY HDs. Considering these taxa and those in Table 2 associated with one of the two methods, in general the taxa closely associated with the EPA samplers preferred slower flow, while those associated primarily with the NY samplers preferred a faster flow. The NY HDs were situated higher in the water column. Perhaps flow could be higher near the water surface than near the sediment, although we do not have any comparative flow measurements. Between the boat action and the effects of wind on the surface, at least more wave action could reasonably be expected near the surface, which might be attractive to taxa that prefer more flow as part of their habitat. In future studies, co-deployment of HDs near the sediment and in the water column (at places where there is significant water depth) may be useful to explore. Co-locating HDs near the sediment and higher in the water column and aggregating the taxa richness data from the two HD location at each station has the potential to retrieve more taxa per station, and therefore to provide a more complete characterization of the macroinvertebrate communities.

Regarding the abundance and density metrics, there might be a greater abundance expected on the EPA HDs might be expected based on their much greater surface area, if the density on the plates of each type of HD was the same. However statistically higher abundance was found on the EPA HDs at only 2 of the 8 stations (Table 3). Higher density was associated with only one method, the NY HD method, but at only 2 of the 8 stations (Figure 4, Table 3). While these results do not clearly indicate higher density on the NY HD plates, they suggest that something other than (or in addition to) surface area could be affecting numbers of macroinvertebrates on the samplers. Besides the different locations in the water column, other potential causal factors for the differences seen in density include differences in plate size and plate spacing. Studies have found that different macroinvertebrate taxa have preferences for different substrate particle sizes [33] [34] [35] [36], which would imply a possible preference for different size spaces. For instance, it might be that some macroinvertebrate taxa find the larger spaces between the NY plates advantageous for colonization. Plate spacing could also affect the level of predation of the communities on the plates. Dudgeon [37] in a study focused on how plate spacing affected predation, found that when fish were excluded, density was greater on the multi-plate samplers with wide spacing.

## 5. Conclusion

The comparability of water quality categories derived from multi-metric and tolerance-based indices, as well as total taxa and EPT taxa richness, all generated from macroinvertebrate community data retrieved by two significantly different Hester-Dendy sampling methods, provide evidence of the robustness of multi-plate sampling methods. There were some statistically significant differences seen in some index and metric values, and community differences by method from the multivariate analyses. The differences that were seen between communities are likely due mainly to the difference in position in the water column between the two methods, though between-plate spacing and plate size may contribute. Multivariate ordinations showed that the location differences in communities were much greater than differences due to sampling method or month. This information, along with site differences shown by selected metrics and indices, indicates that either type of multi-plate sampler method can detect differences in water quality even in a set of stations whose land use all include high levels of development.

## Figures and Tables

**Table 1.** Locations of sampling stations, water depths, and their relation to Niagara River (NR).

Station ID	Tributary Name	Lat	Long	Water Depth (m)⋆	Location
NRCY-01A	Cayuga Creek	43.07499	−78.96388	1.3	Where creek intersects with Cayuga Island
NREL-01A	Ellicott Creek	43.02037	−78.87545	3.0	Just before confluence with Near Tonawanda Island.
NRGL-02A	Gill Creek	43.07832	−79.02586	1.9	Just before confluence with NR. Industrial.
NRGL-03A	Gill Creek	43.07886	−79.0258	1.2	Just slightly upstream from NRGL-02A
NRSC-01A	Scajaquada Creek	42.92911	−78.89842	2.5	Just upstream from confluence with NR. Near Squaw Island.
NRTW-01B	Tonawanda Creek	43.0224	−78.8812	5.2	Just downstream from confluence with Ellicott Creek (NREL-01A), and upstream from confluence with NR.
NRTW-02A	Tonawanda Creek	43.01958	−78.85296	3.2	Well upstream from NRTW-01B
NRTM-01A	Two Mile Creek	43.010892	−78.906402	1.1	At confluence with NR. Almost in NR.

⋆ = Average of three measurements; one each in July, August, and September.

**Table 2.** Results of an Indicator Species Analysis using PC-ORD showing taxa significantly associated (*p* < 0.05) with each HD method. Tolerance Values (TV) and Functional Feeding Group (FFG) from Mandaville (2002).

Taxon	HD method	p value	Number Samples	TV	Feeding Habit/FFG
*Ablabesmyia* sp.	EPA	0.0082	18	8	PRD
*Chironomus* sp.	EPA	0.0002	15	10	CG
*Dicrotendipes* sp.	EPA	0.0088	41	8	CG
*Paratendipes* sp.	EPA	0.028	8	6	CG
*Phaenopsectra* sp.	EPA	0.0026	11	7	SCR
*Tribelos* sp.	EPA	0.002	13	7	CG
*Hydroptila* sp.	EPA	0.035	7	6	SCR
*Caecidotea* sp.	EPA	0.0084	22	8	CG
*Cambaridae*	EPA	0.024	6	6	CG

FFGs: PRD = predator, CG = Collector-Gatherer, SCR = Scraper.

**Table 3.** Results of NYSDEC BAP index scores by month, calculated from macroinvertebrate communities retrieved by EPA and NY HD methods. Highest monthly values in **bold**.

EPA-BAP									
	NRCY-01A	NREL-01A	NRGL-02A	NRGL-03A	NRSC-01A	NRTW-01B	NRTW-02A	NRTM-01A	AVG.

July	4.05	**4.25**	2.65	**4.26**	3.56	**8.06**	4.72	3.3	4.36
August	**5.1**	2.49	2.64	2.59	4.21	7.5	6.87	2.51	4.24
Sept.	4.43	2.31	**5.04**	3.73	**5.75**	7.53	**7.94**	**4.23**	**5.12**
Avg.	4.53	3.02	3.44	3.53	4.51	7.70	6.51	3.35	3.35
SE	0.307	0.619	0.798	0.493	0.649	0.182	0.947	0.497	0.561

NY-BAP									
	NRCY-01A	NREL-01A	NRGL-02A	NRGL-03A	NRSC-01A	NRTW-01B	NRTW-02A	NRTM-01A	AVG.

July	4.04	NV	3.56	4.98	3.84	**7.6**	3.35	3.72	4.44
August	2.14	2.18	2.69	2.35	**3.88**	5.65	5.66	3.11	3.46
Sept.	**5.25**	**2.49**	**5.15**	**5.00**	2.89	6.33	**7.4**	**5.06**	**4.95**
Avg.	3.81	2.34	3.80	4.11	3.54	6.53	5.47	3.96	3.96
SE	0.905	0.155	0.720	0.880	0.324	0.571	1.173	0.576	0.663

NV = no value, SE = standard error.

Samplings where the 3 methods gave different impact category. (allowing for ±0.1 variability from the calculated values) indicated by shading.

**Table 4.** Results of paired (NY vs. EPA) comparisons at each of the 8 study stations for several metrics and indices (1-tailed paired t-tests).

	Abundance	Density	# Taxa	# EPT Taxa	# Insect Taxa	# C-F Taxa	# Tol C-G Taxa	HBI	HBI_10_	MTV	% Tol C-G Indiv.	BAP
*Significant. Difference?*	Yes	Yes	No	No	Yes	No	No	Yes	Yes	Yes	Yes	Yes
*Stations w/Diff.*	NRCY-01A NRSC-01A	NRGL-02A NRTW-01B	none	none	NRTW-01B	none	none	NRTW-02A NRTM-01A	NRTW-01B	NRTW-02A	NRCY-01A NRGL-03A NRTM-01A	NRTW-01B NRTW-02A NRTM-01A
*HD Type* ⋆	EPA	NY	NA	NA	EPA	NA	NA	NY	NY	NY	NY(2), EPA(1)	EPA(2), NY(1)

Signif. Diff. = statistically significant difference between methods. Stations w/Diff. = stations with significant differences between methods, either significant (*p* ≤ 0.05) or highly significant (*p* < 0.01; station IDs in *bold italic*). Tolerant = tolerance values 7 - 10.

⋆ HD type/method with the value(s) indicating better water quality.
